# A Rare Co-association of Autoimmune Thyroiditis and Idiopathic Retroperitoneal Fibrosis

**DOI:** 10.7759/cureus.30980

**Published:** 2022-11-01

**Authors:** Ramesh Adhikari, Akshat Banga, Thoyaja Koritala, Naresh Dasari, Vishwanath Pattan

**Affiliations:** 1 Hospital Medicine, Franciscan Health, Lafayette, USA; 2 Geriatrics, Brown University, Providence, USA; 3 Internal Medicine, Sawai Man Singh Medical College, Jaipur, IND; 4 Internal Medicine, Mayo Clinic Health System, Mankato, USA; 5 Internal Medicine, Roger Williams Medical Center, Cranston, USA; 6 Endocrinology, Upstate University Hospital, Syracuse, USA

**Keywords:** fibroinflammatory, igg4-related disorders, rpf, igg4 disease, autoimmune, steroids, idiopathic retroperitoneal fibrosis, obstructive hydronephrosis, autoimmune thyroiditis

## Abstract

Retroperitoneal fibrosis is a rare fibroinflammatory disease that is reported to be associated with other autoimmune conditions. Here, we report the case of a 51-year-old Caucasian female with a history of autoimmune thyroiditis and Hashimoto’s hypothyroidism who presented with symptoms of fever, chills, and hot flashes for three weeks associated with nausea, vomiting, frequent thirst, and frequent urination. On examination, the patient had elevated blood pressure and an excoriated rash on the forearms. Laboratory evaluation showed elevated blood urea nitrogen and creatinine with a hypertensive emergency. Renal ultrasound showed bilateral hydronephrosis suggestive of obstructive uropathy. Computerized tomography of the abdomen and pelvis was suggestive of extensive retroperitoneal fibrosis. The patient was diagnosed with idiopathic retroperitoneal fibrosis without an identifiable secondary cause. Treatment was focused on relieving the ureteral obstruction, managing renal functions, and optimizing blood pressure, following which immunomodulatory agents were used.

## Introduction

Retroperitoneal fibrosis is a rare fibroinflammatory disease that can be idiopathic or secondary to known causes such as drugs, infection, malignancy, radiation, infection, or surgery [1]. The majority of the reported retroperitoneal fibrosis cases are termed idiopathic (>70%) but often arise in patients with other autoimmune conditions [2]. Idiopathic retroperitoneal fibrosis (IRF) commonly presents as obstructive uropathy between 50 and 60 years of age [1,2]. Here, we report a rare co-association of autoimmune thyroiditis and IRF in a 51-year-old Caucasian female who presented with obstructive uropathy without any identifiable secondary causes.

## Case presentation

A 51-year-old postmenopausal caucasian female with a medical history significant for hypothyroidism due to Hashimoto’s, hypertension, and remote history of illicit drug use presented to the emergency department with a chief complaint of “not feeling well.” She was transferred to our hospital for acute renal failure of unknown cause.

Prior to admission, the patient had a three-week history of fever, chills, hot flashes, associated nausea, vomiting, and an itchy rash over the forearms. The patient attributed these symptoms to menopause. However, the symptoms progressively worsened, and she presented to the emergency department when persuaded by her husband. Because of financial constraints, the patient was noncompliant with antihypertensive medications and primary physician visits. The patient previously had a history of methamphetamine, opioid, and marijuana use and was homeless a few years ago. She smoked one pack of cigarettes a day and had occasional alcohol intake. The urine drug screen was negative at admission. Home medications included levothyroxine 150 µg daily, but she was not taking the antihypertensive medications. On presentation, vitals showed elevated blood pressure of 180/90 mmHg, heart rate of 78 beats per minute, respiratory rate of 15 breaths per minute, and temperature of 96.9°F. The patient had excoriated rash on both forearms secondary to itching from dry skin. Thyroid was not palpable on physical examination. The rest of the physical examination was unremarkable.

**Figure 1:**
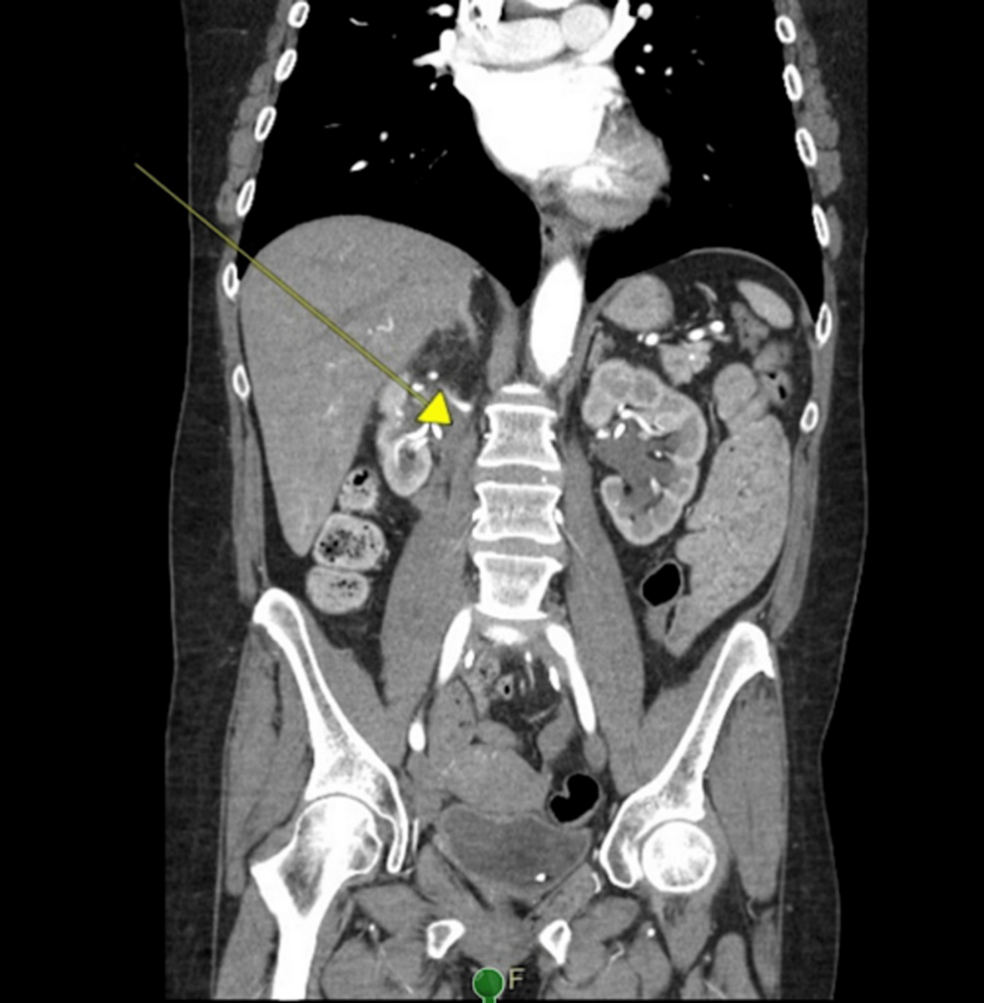
Retroperitoneal fibrosis tissue obstructing the right ureter causing hydronephrosis.

**Figure 2:**
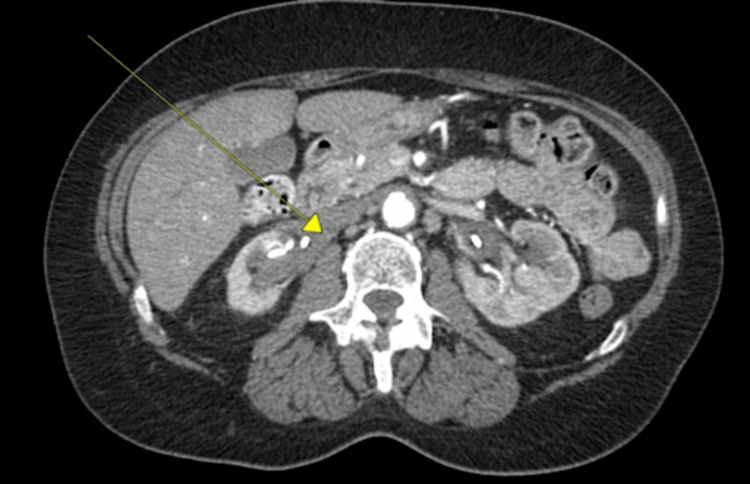
Right-sided retroperitoneal fibrosis.

Urinalysis and blood cultures did not identify any infectious etiology. Renal ultrasound showed bilateral hydronephrosis suggestive of obstructive uropathy. Computed tomography angiography (CTA) of the chest, abdomen, and pelvis later showed more right ureteric fibrosis than left and abnormal narrowing in the rectosigmoid region (Figures 1, 2). The patient underwent colonoscopy for the rectosigmoid narrowing, which showed polyps in ascending, mid-transverse, and descending colon between 3 mm and 10 mm in size, and no anatomical narrowing of the rectosigmoid region (pathology showed one tubulovillous adenoma and the rest are tubular adenomas with no evidence of high-grade dysplasia).

Autoantibodies by multiplex flow immunoassay with a reflex display of antigens-dsDNA, chromatin, ribosomal P, SS/A, SS/B, Sm, Sm RNP, RNP, Scl 70, Jo 1, and centromere B were negative. IgG4 levels were negative. Anti-neutrophil cytoplasmic autoantibody (ANCA), myeloperoxidase antibody, and serine proteinase antibodies were negative. Flow cytometry and peripheral blood smear to rule out leukemia, and lymphoma showed no evidence of acute leukemia, monoclonal B-lymphocyte, or aberrant T-lymphocyte phenotype (Table 1).

**Table 1:** Laboratory values. eGFR: estimated glomerular filtration fate; ESR: erythrocyte sedimentation rate; CRP: C-reactive protein; PCR: polymerase chain reaction; Ag: antigen; AB: antibody

Diagnostic test	Initial lab values	Normal values
Sodium Na^+^	137 meq/L	133–144 meq/L
Potassium K^+^	6.1 meq/L	3.5–5.1 meq/L
Chloride	103 meq/L	98–107 meq/L
Carbon dioxide	14 meq/L	21–31 meq/L
Anion gap	20 meq/L	6.2–14.7 meq/L
Blood urea nitrogen	107 mg/dL	7–25 mg/dL
Creatinine	12.06 mg/dL	0.60–1.20 mg/dL
eGFR	3	60–9,999
Calcium	8.9 mg/dL	8.6–10.3 mg/dL
Glucose	93 mg/dL	70-99 mg/dL
Phosphorous	10.6 mg/dL	2.5–4.5 mg/dL
Creatinine 24-hour urine	922 mg	500–1,400 mg (24 hours)
Creatinine clearance 24-hour urine	14.6 mL/minute	71.0–151.0 mL/minute
Anti-thyroid peroxidase antibody	290.46 IU/mL	0–9 IU/mL
Thyroglobulin antibody	46 IU/mL	0–4 IU/mL
Thyroid-stimulating hormone	0.030 µIU/L	0.27–4.20 µIU/mL
Parathyroid hormone	462.2 pg/mL	12–88 pg/mL
Vitamin D	8.0 ng/mL	30.0–100.0 ng/mL
IgG subclass 4	80 mg/dL	1–123 mg/dL
Lymphocytes AB CT	1.4 10^3^/μL	0.6–3.4 10^3^/μL
Anti-neutrophil cytoplasmic antibody IgG	<1:20	<1:20
Myeloperoxidase antibody IgG	0	0–19
Serine protease 3 IgG	1	0–19
ESR	37 mm/hour	0–30 mm/hour
CRP	0.9 mg/dL	0.0–0.5 mg/dL
Histoplasma antigen aerum	Not detected	Not detected
COVID-19 PCR	Negative	Negative
Influenza A	Negative	Negative
Influenza B	Negative	Negative
HIV Ag/Ab screen	Negative	Negative
Quantiferon gold plus	Negative	Negative
Hepatitis A IgM	Non-reactive	Non-reactive
Hepatitis B surface Ag	Non-reactive	Non-reactive
Hepatitis B Core Ab IgM	Non-reactive	Non-reactive
Hepatitis B Surface Ab	<3.1mIU/mL	<10.0 mIU/mL
Hepatitis C Ab, IgG	Non-reactive	Non-reactive
dsDNA, chromatin, ribosomal P, SS/A, SS/B, Sm, SmRNP, RNP, Scl 70, Jo 1, and centromere B Ab	Negative	Negative

Lactate dehydrogenase levels were normal. Erythrocyte sedimentation rate of 37 mm/hour (0-30 mm/hour) and CRP levels of 0.9 mg/dL (0.0-0.5 mg/dL) were elevated at presentation (Table 1). COVID-19 rapid and PCR tests, influenza A and B, Histoplasma Ag, gold QuantiFERON, HIV, and hepatitis profile were negative (Table 1). During hospitalization, the patient transiently complained of blurry vision in one eye. Magnetic resonance imaging (MRI) of the brain showed old lacunar infarcts, and the symptoms resolved in a few minutes. Retroperitoneal node biopsy was not performed as it was not accessible for CT-guided biopsy.

The patient denied abdominal surgeries or a history of malignancy or radiation therapy. The diagnosis of IRF was made.

She had pre-existing chronic Hashimoto’s or autoimmune thyroiditis with anti-thyroid peroxidase antibody (TPO) 290.46 IU/L (high) (0-9 IU/mL), thyroglobulin antibody-46 units (high) (0-4 IU/mL), thyroid-stimulating hormone (TSH) 0.030 µIU/L (low) (0.27-4.20 µIU/mL), and TSH immunoglobulin <0.10 (<=0.54 IU/L) (Table 1). The patient presented with a suppressed TSH, which could represent an overdose of her levothyroxine therapy or perhaps sick euthyroid in the setting of acute illness (free T4 was not assessed). She reportedly increased her dose from 100 µg to 150 µg about six months ago, which is likely the cause of the lower TSH. Her levothyroxine dose was decreased to 125 µg at the time of discharge, and she asked for a follow-up. Follow-up TSH 0.250 µIU/mL (0.27-4.20 µIU/mL) and T3 1.34 (0.55-1.60 ng/dL) done four months later normalized.

Because IRF is also considered a part of the IgG4-related disease spectrum, the patient was started on 1 mg/kg prednisone and asked to follow up in four weeks for a follow-up MRI of the abdomen and pelvis. The patient will have her ureteral stents removed in four weeks. She was counseled to quit smoking. At the time of discharge, the patient was off hemodialysis and was asked to follow up at an outpatient with nephrology for further care.

## Discussion

Our patient is a 51-year-old postmenopausal woman who presented with acute kidney injury with obstructive uropathy, hypertensive emergency with hyperkalemia, and imaging evidence suggestive of retroperitoneal fibrosis. The patient was not using any medication or drugs known to cause retroperitoneal fibrosis. She did not have any evidence of infectious etiology or malignancy. Work-up for IgG4-related autoimmune disease was negative. The patient never had any intra-abdominal surgeries or radiation therapy. She was diagnosed with IRF during the hospitalization.

IRF is considered to be a fibroinflammatory disease typically arising around the aorta and iliac arteries, hallmarked by fibrosis and chronic inflammation histologically [2]. It presents extensive retroperitoneal fibrosis and renal dysfunction due to ureteric obstruction [2]. IRF is believed to be a part of the IgG4-related autoimmune disease spectrum [2]. Clinically, it has been seen that having one autoimmune condition increases the risk of having another [3]. Autoimmune diseases such as autoimmune thyroiditis have been shown to be associated with fibroinflammatory complications [2]. However, this association is rare and not causal. Ceresini et al. (2015) monitored thyroid lab panels in 73 consecutive patients with new-onset IRF and found a significantly higher prevalence of elevated anti-TPO Ab in IRF patients compared to the controls [4].

IRF presents with non-specific systemic symptoms, including pain (usually limited to the abdomen, back, or flank), anorexia, weight loss, and fatigue [5,6]. Ureteral involvement is the most common complication leading to ureteric obstruction, presenting with ureteric colic-like pain and urologic manifestations such as hydrocele, varicocele, retrograde ejaculation, and erectile dysfunction [5,6]. CT scan is the diagnostic modality of choice to diagnose IRF, as seen in our case, followed by MRI [5,6]. However, F‑fluorodeoxyglucose positron emission tomography and invasive procedures such as retroperitoneal biopsy are sometimes performed as well [7].

The management of retroperitoneal fibrosis focuses on alleviating symptoms and relieving ureteric obstruction to restore renal functions, along with management of the cause, if any [2,8]. Depending on the severity of the disease and obstruction, management can be conservative (medical) [9] or invasive (surgical) [8]. Relieving ureteric obstruction remains the first goal of IRF management. As seen in our case report, conservative treatment with double-J stenting followed by medical therapy remains the preferred approach [2]. Because IRF is part of the IgG4-related disease spectrum, immunomodulation with glucocorticoids is the usual first approach. The glucocorticoid-resistant disease may be treated with immunosuppressive drugs such as methotrexate and mycophenolate with or without prednisone, but the evidence for this practice is limited to uncontrolled clinical trials. Recent evidence has shown promise with biologics such as rituximab and tocilizumab in treating IRF [10,11].

## Conclusions

The co-association of autoimmune thyroiditis and idiopathic retroperitoneal fibrosis is rare, but the relationship is not causal. IRF is an IgG4 spectrum disorder, and glucocorticoids are the usual first-line agents. The glucocorticoid-resistant disease may be treated with immunosuppressive drugs and, finally, as a last resort, biologic agents such as rituximab and tocilizumab. Hence, evaluating autoimmune thyroiditis and hypothyroidism is reasonable for patients diagnosed with IRF.
